# Efficient Chitosan/Nitrogen-Doped Reduced Graphene Oxide Composite Membranes for Direct Alkaline Ethanol Fuel Cells

**DOI:** 10.3390/ijms22041740

**Published:** 2021-02-09

**Authors:** Selestina Gorgieva, Azra Osmić, Silvo Hribernik, Mojca Božič, Jurij Svete, Viktor Hacker, Sigrid Wolf, Boštjan Genorio

**Affiliations:** 1 Faculty of Mechanical Engineering, University of Maribor, Smetanova 17, 2000 Maribor, Slovenia; selestina.gorgieva@um.si (S.G.); silvo.hribernik@um.si (S.H.); 2 Faculty of Electrical Engineering and Computer Science, University of Maribor, Koroška cesta 46, 2000 Maribor, Slovenia; azra.osmic@um.si; 3 Dravske Elektrarne Maribor d.o.o., Obrežna ulica 170, 2000 Maribor, Slovenia; mojca.bozic@dem.si; 4 Faculty of Chemistry and Chemical Technology, University of Ljubljana, Večna pot 113, 1000 Ljubljana, Slovenia; jurij.svete@fkkt.uni-lj.si; 5 Institute of Chemical Engineering and Environmental Technology, Graz University of Technology, Stremayrgasse 9, 8010 Graz, Austria; viktor.hacker@tugraz.at (V.H.); sigrid.wolf@tugraz.at (S.W.)

**Keywords:** chitosan, nitrogen-doped reduced graphene oxide, anion-exchange membrane, direct alkaline ethanol fuel cell

## Abstract

Herein, we prepared a series of nanocomposite membranes based on chitosan (CS) and three compositionally and structurally different N-doped graphene derivatives. Two-dimensional (2D) and quasi 1D N-doped reduced graphene oxides (N-rGO) and nanoribbons (N-rGONRs), as well as 3D porous N-doped graphitic polyenaminone particles (N-pEAO), were synthesized and characterized fully to confirm their graphitic structure, morphology, and nitrogen (pyridinic, pyrrolic, and quaternary or graphitic) group contents. The largest (0.07%) loading of N-doped graphene derivatives impacted the morphology of the CS membrane significantly, reducing the crystallinity, tensile properties, and the KOH uptake, and increasing (by almost 10-fold) the ethanol permeability. Within direct alkaline ethanol test cells, it was found that CS/N rGONRs (0.07 %) membrane (P_max_. = 3.7 mWcm^−2^) outperformed the pristine CS membrane significantly (P_max_. = 2.2 mWcm^−2^), suggesting the potential of the newly proposed membranes for application in direct ethanol fuel cells.

## 1. Introduction

The use of environmentally friendly materials to circumvent the steady depletion of fossil fuels, the reduction of global pollution, and electricity consumption, are among the most investigated topics of scientific and technological research in recent decades [1]; all of the above is covered in energy conversion devices, i.e., fuel cells. These are devices that convert chemical energy directly to electrical energy using a fuel that is oxidized (such as natural gas, hydrogen, ethanol, methanol, formic acid, or phosphoric acid), and an oxidizing agents (such as air or oxygen) [2]. As highly efficient, benign, and environmentally friendly devices, fuel cells have been identified as promising and potent technologies for zero emission, electrochemical energy conversion, and power generation, which, apart from their considerable maturation over the last decade, are still constrained by technological barriers, such as insufficient durability and cell life, as well as the high cost of fuel cell components that hamper their commercialization.

For this reason, critical attention has focused increasingly on direct alkaline ethanol fuel cells (DAEFC), where non-noble metal catalysts can be used, together with widely available ethanol, a fuel produced from biomass feedstocks. DAEFCs convert chemical energy stored in liquid ethanol directly into electricity. Non-noble metal cathodes (e.g., perovskite) are robust, and exhibit high tolerance to impurities and fuel crossover through the membrane. The main part of the DAEFC is the membrane-electrode assembly (MEA), which is formed by sandwiching the multilayer structure of an anode diffusion layer, an anode-catalyst layer, the anion exchange membrane (AEM), a cathode-catalyst layer and a final cathode diffusion layer. The function of the membrane is to conduct OH- (hydroxide) ions from the cathode to the anode, and to separate the anode and cathode electrodes. However, the development of AEM DAEFC suffers from the limitations of the low thermal and chemical stability of AEMs. Since the mobility of hydroxide is only about 57% of the mobility of a proton, a high ion exchange capacity (IEC) is required to achieve high ion conductivity compared to proton exchange membranes (PEMs) [3]. Currently, commercially available AEMs are based on quaternary ammonium exchange groups, and they are developed by Japan Tokuyama Corporation (Tokyo, Japan) on an unspecified backbone polymer (A201 membrane), Solvay (Beveren, Belgium) on a cross-linked fluorinated polymer (Morgan ADP membrane) and Fumatech, Bietigheim-Bissingen, Germany on polysulfones (FAA membrane) [4].

Synthesis of synthetic polymer AEMs is often complex, environmentally unfriendly, and not cost-effective. Currently, the trend towards bioeconomy and bio-based resource utilization, gives momentum to naturally derived materials also in fuel cells’ application, as a part of MEA. One of the most preferred polysaccharide-based biomaterials, in addition to cellulose, is chitosan (CS). CS is non-toxic and thermally stable polymer already found in a prominent place in membranes’ development for fuel cells’ application. CS is also a biobased polymer, obtained mainly from the exoskeletons of crustaceans and the mycelium of some fungi, which gives it an advantage in production due to its low cost and environmental aspects [5,6]. Chitosan as a nitrogen-containing biomass has received much attention for the preparation of nitrogen-doped carbon materials for the oxygen reduction reaction in fuel cells and other electrochemical devices [7]. Vaghari et al. [8] investigated the ionic conductivity of pure CS membranes with different degrees of deacetylation and molecular weight and suggested their suitability for use in alkaline fuel cells. The main flaw of CS, however, is its poor ionic conductivity, which is usually compensated by chemical modification with other functionalities. CS consists of 2-amino-2-deoxy-(1,4)-β-d-glucopyranose units and has a hydrophilic character due to the amino (-NH_2_) and hydroxyl (-OH) groups, both of which allow chemical modification. Recently, CS was modified to form derivatives in which the quaternary ammonium groups are attached [9]. The addition of inorganic components also improved ion conductivity and contributed to the thermal-mechanical properties of the final composite membrane. The introduction of graphene oxide (GO) has been identified to improve mechanical stability, prevent fuel crossover and swelling of the membrane, and improve ionic conductivity due to the π–π interaction between the aromatic chain of reduced GO (rGO) and the polymer backbone. Strong hydrogen bonds and electrostatic attraction between negatively charged GO plates and positively charged polysaccharide CS groups make CSGO a stable nanocomposite with excellent mechanical and thermal properties [10].

Depending on the type of ionic conductivity within the structure of the polymers, AEMs are divided into polyelectrolyte- consisting essentially of ionomer, and alkali-doped polymer membranes, consisting of an electronegative heteroatom as nitrogen. In recent years, a variety of AEMs have been prepared by the introduction of quaternary ammonium (QA), imidazolium, guanidinium, phosphonium groups, or permethyl cobaltocenium into various polymer backbones, such as poly(styrene), poly(vinyl alcohol), poly(phenylene oxide), poly(arylene ether ketone), and poly(arylene ether sulfone) [11]. In a previous article [12], our group used GO with oxygen functional groups (epoxy, carboxy, keto, hydroxy, etc.), which are similar to poly(styrene), poly(vinyl alcohol), poly(phenylene oxide), and poly(arylene ether ketone).

The presented work demonstrates the processing and introduction of three, compositionally and structurally different types of N-doped graphene derivatives into a CS membrane, forming a self-standing, organic-inorganic nanocomposite membrane. Herein, we hypothesize that introduction of two-dimensional (2D) and quasi 1D N-doped reduced graphene oxides (N-rGO) and 3D porous N-doped graphitic particles with nitrogen functional groups: pyridinic N, pyrrolic N, and quaternary or graphitic N within CS membrane, will enhance its ultimate performance in alkaline fuel cells. The comprehensive study was applied to assess the physicochemical integration between components, the micro structuring, mechanical profile, ethanol permeability, and alkaline uptake, as well as membranes feasibility within application-relevant cell performance test.

## 2. Materials and Methods

### 2.1. Materials

Graphite (Imerys, Timrex PP44, Bodio, Switzerland), multi-walled carbon nanotubes (MWCNTs )(NanoTechLabs C-Grade MWNTs, Yadkinville (NC), USA), H_2_SO_4_ (Sigma-Aldrich, Darmstadt, Germany, ACS reagent, 95–98%), H_3_PO_4_ (Sigma-Aldrich, Darmstadt, Germany, ACS reagent, ≥85 wt.% in H_2_O), KMnO_4_ (Sigma-Aldrich, Darmstadt, Germany, ACS reagent, ≥99%), H_2_O_2_ (Sigma-Aldrich, Darmstadt, Germany, contains inhibitor, 30 wt.% in H_2_O, ACS reagent), HCl (Sigma-Aldrich, Darmstadt, Germany, ACS reagent, 37%), chitosan (CS, degree of deacetylation: 90%, molecular weight: 50–100 kDa) was purchased in powder form with particle sizes below 200 µm from Biolog Heppe GmbH, Landsberg, Germany. Magnesium hydroxide (Mg(OH)_2_) nanopowder, sodium hydroxide (NaOH), ethanol (EtOH) and potassium hydroxide (KOH) were purchased from Sigma-Aldrich, Darmstadt, Germany. All chemicals were used as received. For the single cell test, the following materials were used: KOH (Sigma Aldrich, 1.0 M Fixanal 1 L Ampoule), 2-Propanol (Roth, 99.9% p.a.), anion exchange ionomer (Fumion FAA-3 solution in NPM, 10%, Bietigheim-Bissingen, Germany), PtRu/C catalyst (HiSPEC 10000, Platinum, nominally 40%, Ruthenium, nominally 20% on carbon black, Johnson Matthey, London, UK).

### 2.2. Preparation of Graphene Oxide (GO) and Graphene Oxide Nanoribbons (GONRs)

GO and GONRs were synthesized according to a slightly modified previously published method [13]. A mixture of concentrated H_2_SO_4_/H_3_PO_4_ (vol. ratio = 9:1) (1000 mL:111 mL) was added to the graphite (Imerys, Timrex PP44) (20 g) or MWCNTs (NanoTechLabs C-Grade MWNTs) (20 g) to a mixture of concentrated H_2_SO_4_/H_3_PO_4_ (vol. ratio = 9:1) (600 mL:67 mL) and then KMnO_4_ in aliquots (6 or 7 aliquots × 20 g) was added while stirring, producing a slight exotherm to 35–40 °C. The reaction mixture was then stirred using a mechanical stirrer at room temperature in an open 3 L beaker for 10 days. The reaction mixture was then poured onto ice (800 mL or 1000 mL) and 30 vol.% H_2_O_2_ (15 or 20 mL) was added dropwise until the color changed from purple to yellow. For workup, the mixture was transferred to plastic centrifuge bottles, diluted with H_2_O, and centrifuged (4100 rpm for 30 min). The supernatant was decanted away and the remaining solid was washed with H_2_O and centrifuged (4100 rpm for 30 min). After the supernatant was removed, the remaining material was washed and centrifuged (4100 rpm for 90 min) in succession with 10% HCl and H_2_O. After this multiple wash, the remaining material was freeze-dried and stored at room temperature.

### 2.3. Polyenaminone (4ca)

Poly(2-(3-aminopropenoyl)-6-{3-[(1,3-phenylene)amino]propenoyl}pyridine) (4ca) was prepared following the general literature protocol for the synthesis of a related polyenaminones [14]. A mixture of (2*E*,2’*E*)-1,1’-(pyridine-2,6-diyl)bis(3-(dimethylamino)prop-2-en-1-one) (2c) (273 mg, 1 mmol), 1,3-phenylenediamine dihydrochloride (3a) (181 mg, 1 mmol), and methanol (10 mL) was shaken on an orbital shaker (400 r.p.m.) at room temperature for 96 h. The precipitate was collected by filtration, washed with methanol (2 mL), and air-dried to give 4ca as an orange solid in a quantitative yield.

### 2.4. Nitrogen-Doped Reduced Graphene Oxide (N-rGO) and Nitrogen-Doped Reduced Graphene Oxide Nanoribbons (N-rGONRs)

GO and GONRs were heat treated in an NH_3_ atmosphere (constant flow 30 mL/min) in a quartz tube by using the following heat treatment protocol: (i) room temperature to 800 °C (heating rate 10 K/min); (ii) hold at 800 °C for 10 min; (iii) 800 °C to room temperature (cooling rate 20 K/min). The products of pyrolysis in NH_3_ were labelled as N-rGO and N-rGONRs.

### 2.5. Pyrolysis of Polyenaminone (4ca) to Graphitic N-Polyenaminone (N-pEAO)

Polyenaminone (4ca) was heat treated in an N_2_ atmosphere (constant flow 30 mL/min) in a quartz tube by using the following heat treatment protocol: (i) room temperature to 800 °C (heating rate 10 K/min); (ii) hold at 800 °C for 10 min; (iii) 800 °C to room temperature (cooling rate 20 K/min). The product of pyrolysis was labelled as N-pEAO.

### 2.6. Preparation of CS and CS/N-rGO(rGONRs, pEAO) Membranes

CS membranes were prepared out of 1 wt.% CS solution in Milli-Q with the addition of 1 M HCl to pH = 2–2.5. Under constant stirring, the CS was completely dissolved, and to obtain CS neutralization, the pH was again raised to 6–6.5 by adding 1 wt.% Milli-Q water dispersion of magnesium hydroxide (Mg(OH)_2_). Final dispersion was allowed to stir at 500 min^−1^ overnight to allow the pH stabilization (pH~6). Membranes were prepared by pouring the 25 mL dispersion into petri dishes with d = 90 mm and left to air-dry. The completely dry membranes were neutralized with 1 M NaOH and then washed several times with distilled water and left to re-dry in air.

For preparation of the CS/N-rGO membranes, the 1 wt.% N-rGO, pre-dispersed in Milli-Q water was added to 25 mL of 1% CS dispersion, to final concentration of 0.01%, 0.04% and 0.07% w/v. Final dispersion was vortexed at 2500 min^−1^ to obtain a homogeneous dispersion. CS/N-rGO membranes were processed further following the same procedures as described for neat CS membranes, and all membranes were fridge-stored until the following characterizations.

### 2.7. Characterization

#### 2.7.1. X-ray Photoelectron Spectroscopy (XPS) Analysis

XPS measurements were performed using a SPECS PHOIBOS 150 Hemispherical Energy Analyzer, Berlin, Germany, with a monochromate Al *Kα* X-ray source. Survey spectra were measured using a pass energy of 40 eV at a resolution of 0.2 eV step^−1^ and a total integration time of 0.2 s point^−1^. Core-level spectra were measured using a pass energy of 20 eV at a resolution of 0.05 eV step^−1^ and a total integration time of 0.5 s point^−1^. Deconvolution was performed using CasaXPS software (http://www.casaxps.com/) with a Shirley-type background and 70–30 Gaussian–Lorentzian peak shapes. In general, spectra were charge referenced using the position of the C*1s* at 284.0 eV.

#### 2.7.2. Scanning Electron Microscopy (SEM) and Energy-Dispersive X-ray Spectroscopy (EDS) Analysis

Microstructure characterization of N-rGO and N-rGONRs and N-pEAO was performed by a scanning field emission electron microscope, Zeiss ULTRA plus SEM – Jena, Germany. Samples were adhered to the conductive carbon tape placed on an aluminum SEM holder. SEM images were taken at 2 kV using an SE2 detector at WD 5.5 mm. Further, elemental analysis of the samples was done inside SEM using EDS analysis with an Oxford X-Max SDD detector - High – Wycombe, UK, with working surface area of 50 mm^2^, processed with INCA software - Wycombe. EDS analysis was done at 20 kV.

For visualization of membranes’ microstructure, the field emission (FE)-SEM analysis was performed using a Carl Zeiss FE-SEM SUPRA 35 VP electron microscope. Imaging was performed at 1 kV accelerating voltage at an approximately 4.5 mm working distance. The membranes were attached to aluminum sample holders via conductive carbon adhesive tape. Prior to analysis, a layer of palladium was sputtered on the surface of membrane samples.

#### 2.7.3. Brunauer–Emmett–Teller (BET) Analysis

Specific surface area (m^2^/g) was measured by N_2_ adsorption at 77 K on an ASAP 2020 Micromeritics instrument, Norcross, GA, USA, by using the Brunauer–Emmett–Teller (BET) analysis method. Samples were degassed under vacuum (5 millitorrs) at 120 °C for 2 h.

#### 2.7.4. Evolved Gas Analysis (EGA) by Thermogravimetric Analysis/Mass Spectrometry (TGA–MS)

Thermogravimetric measurements were performed on a Netzsch 449 F3 Jupiter, Selb, Germany, instrument under a dynamic Ar (5.0) flow with a flow rate of 60 mL min^−1^ in a temperature range from 30 °C to 900 °C. A heating rate of 10 K min^−1^ was used. About 15 mg of sample was placed in an alumina (0.3 mL Al_2_O_3_) crucible. Simultaneously, mass spectrometry was performed on an MS 403C Aëolos with an SEM Chenneltron detector, Selb, Germany, and system pressure of 2 × 10^−5^ mbar. Gases that evolved under TG heat treatment were transferred to the mass spectrometer through a transfer capillary, quartz ID 75 μm, which was heated up to 220 °C. The upper limit of the mass spectrometer detector was 100 AMU.

#### 2.7.5. C, H, N Elemental Analysis

Microanalyses were performed by combustion analysis on a Perkin-Elmer CHN Analyzer 2400 II, Rodgau, Germany.

#### 2.7.6. ζ Potential Measurement

The ζ potential measurements were conducted on N-rGO, N-rGONRs, and N-pEAO dispersions, using a Nano ZS ZEN360 (Malvern Instruments Ltd., Malvern, UK). Each dispersion was prepared at a concentration of 0.001 wt.%, using Milli-Q water as a dispersant, applying intensive stirring and 1 h ultrasonication. The ζ potential values were obtained over the pH range of 2 to 12. The average values and Standard Deviations were calculated from at least three individual measurements.

#### 2.7.7. Attenuated Total Reflectance—Fourier Transform Infrared (ATR-FTIR) Spectroscopy

ATR-FTIR spectra were obtained on a Spectrum One FTIR spectrometer (Perkin-Elmer) with a Golden Gate ATR attachment and a diamond crystal for all components, as well as CS membranes, containing variable concentrations of N-rGO, N-rGONRs, and N-pEAO. The absorbance measurements were recorded within the 650–4000 cm^−1^ region, with 16 scans and a resolution of 4 cm^−1^.

#### 2.7.8. X-ray Diffraction Spectroscopy (XRD)

XRD patterns of the membranes were recorded on a D2 X-ray diffractometer (Bruker Siemens). Reflections at 2*θ* were observed between the range 5° and 70°, with an increment of 0.03°, using a Si holder at a voltage of 30 kV and current of 10 mA. The relative crystallinity degree of the polymer phase was determined by the ratio between the integrated area of the crystalline peaks and the integrated total area of the diffraction spectra [15].

#### 2.7.9. Mechanical Testing

CS/N-rGO, N-rGONRs, and N-pEAO membranes were analyzed by a Shimadzu, AG-X plus 10 kN electromechanical universal testing machine. Membrane samples with specimen dimensions: 10 mm × 20 mm were mounted vertically. The effective clamping distance was 25 mm. Application of a tensile force (10 kN load cell) proceeded at 1 mm/min. Two specimens were tested per sample, and average values and standard deviations were calculated. Prior to testing, the membranes were equilibrated in distilled water for 3 h. The tensile modulus (MPa), tensile strength (MPa) and elastic elongation (%) were calculated from the stress/strain data curves from respective membranes.

#### 2.7.10. KOH Uptake and In-Plane Swelling

KOH uptake and a swelling test of membranes was performed in 6 M KOH at 60 °C. Membrane samples of 1 × 1 cm^2^ were weighted, and their surface areas were measured, before (W_dry_, A_dry_, respectively) and after immersion in 6 M KOH (W_wet_, A_we_t, respectively), where the surplus alkaline solution on the membrane surface was blotted with tissue paper. Measurements were performed after 1 h, 2 h, and 24 h of immersion. Accordingly, KOH uptake (%) and in-plane swelling (%) were determined by the following Equations (1) and (2):

(1) $$KOH\;uptake\;\left(\%\right)=\frac{W_{wet}-W_{dry}}{W_{dry}}\times 100$$

(2) $$SR_{In-plane}=\frac{A_{wet}-A_{dry}}{A_{dry}}\times 100$$

#### 2.7.11. Ethanol Permeability

Ethanol permeability through a 1% CS membrane with N-rGO, N-rGONRs, and N-pEAO was measured in a temperature probe at 25 °C in two temperature-controlled diffusion cells. The membrane, with an area of 66.04 cm^2^, was located between the two temperature-controlled diffusion cells (the volume of each cell unit was 25 mL). Reservoir A was filled with 25 mL of 6 M KOH, reservoir B was filled with 25 mL of 2 M EtOH/6 M KOH (8.5 w/w% ethanol). To create the same pressure on the membrane, we filled both reservoirs at the same time, as this represents the natural working environment of the fuel cell. The ethanol concentration was determined using a conductometer at different time intervals. The ethanol permeability (*P*) was calculated according to Equation (3):

(3) $$P\;\left(\frac{cm^{2}}{s}\right)=\frac{\left(C_{B}-C_{B0}\right)}{\left(t-t_{0}\right)}\times \frac{V_{B}l}{AC_{A0}}$$

where *CA*_0_ is the initial concentration of ethanol in reservoir *B*, and *V_B_* is the volume of KOH in reservoir A. A represents the surface of the membrane and L is the thickness of the membrane.

#### 2.7.12. Cell Performance Measurement (Single Cell Testing, DAEFC Performance with CS-GO Membranes)

The DAEFC performance of the pristine CS, CS/N-rGO, CS/N-rGONRs, and CS/N-pEAO membranes was investigated via single cell tests. Prior to the fuel cell experiments, the membranes were doped with OH- by immersion in 1 M KOH (1.0 M Fixanal 1 L Ampoule, Sigma Aldrich, Darmstadt, Germany) for 24 h and extensive washing with distilled water. Electrodes were developed by depositing ink slurries of the anode/cathode catalysts to the gas diffusion layer (GDL) using an automatic ultrasonic spray coater from Sono-Tek. The catalyst ink was prepared by dispersing the electrocatalysts in a mixture of 2-propanol (99.9% p.a., Roth) and distilled water (7:3) containing 30 wt.% of a commercial anion exchange ionomer (fumion^®^ FAA-3 solution in NPM, 10%). The cathode was prepared by spraying the ink slurry of a commercial PtRu/C catalyst (Platinum, nominally 40%, Ruthenium, nominally 20% on carbon black, HiSPEC^®^ 10000) onto a carbon paper as GDL (Sigracet 29 BC, fuel cell store, 0.235 mm thick), whereas an ink of a PdNiBi/C catalyst synthesized by the modified instant reduction method [16] was sprayed on a carbon cloth (ELAT—hydrophilic plain cloth, fuel cell store, 0.406 mm thick) to fabricate the anode. The electrode preparation resulted in a metal loading of 0.5 mg cm^−2^ for the cathode and 0.75 mg cm^−2^ for the anode. The membrane electrode assemblies (MEAs) with a size of 2 × 2 cm^2^ were prepared by placing the membrane electrolyte between the electrodes. The MEAs were assembled carefully in a self-constructed DAEFC. Pure oxygen gas (5.0, 99.995%) with a constant flow rate of 25 mL min^−1^ was used as a cathode feed gas. A mixture of 1 M ethanol in 1 M KOH served as anode fuel (5 mL min^−1^). The membranes doped with N-rGONRs and N-pEAO were tested additionally in 3 M EtOH/5 M KOH to determine the influence of the fuel concentration on the cell performance. The measurements were generally conducted at room temperature for all graphene doped membranes and a chitosan reference membrane, to investigate the influence of the graphene concentration (0%, 0.01%, 0.04%, 0.07%) in the samples on the DAEFC performance. To evaluate the temperature dependence, experiments were also conducted at higher temperatures (35 °C, 43 °C, 50 °C, and 57 °C). The current densities (I) and cell potentials (V) of the single cells were determined using a Reference 600TM Potentiostat/Galvanostat/ZRA from Gamry Instruments - Warminster, PA, USA. The results were plotted in a current density—potential (I–V) diagram, with additional indication of the power density.

## 3. Results and Discussion

### 3.1. Synthesis and Characterizations of N-rGO, N-rGONRs, and N-pEAO

The resulting properties of polymer/graphene composites can be affected by the type of graphene used, its specific surface area, dispersibility in a polymer matrix, interfacial interactions, surface functionalities, the processing method, etc. [17]. In this respect, we have synthesized 3-representative types of nitrogen doped graphene derivatives, i.e., 2D nitrogen-doped reduced graphene oxide (N-rGO), quasi-1D nitrogen-doped reduced graphene oxide nanoribbons (N-rGONRs), and 3D N-polyenaminone (N-pEAO). Graphene-based materials N-rGO and N-rGONRs were synthesized by a two-step reaction starting using graphite and multi-walled carbon nanotubes (MWCNTs), respectively. The first step was a wet chemistry, top-down approach—a modified Hummers to yield GO and GONRs. GO and GONRs were subsequently reduced and doped with nitrogen in an NH3 atmosphere to yield N-rGO and N-rGONRs. Graphitized N-polyenaminone (N-pEAO) was synthesized in a two-step reaction employing a wet chemistry bottom-up approach. In the first step, polyenaminone (4ca) was synthesized by acid-catalyzed amino–enaminone “click” polymerization reaction [14,18]. Such polymer with inherently layered morphology (Figure S1) was then, in a second step, pyrolyzed in an inert atmosphere to yield graphitic N-polyenaminone (N-pEAO). All three N-doped materials exhibited a highly defected graphene like structure according to Raman spectroscopy (Figure S2). Raman spectra exhibited representative D, G and 2D peaks.

**Scheme 1:**
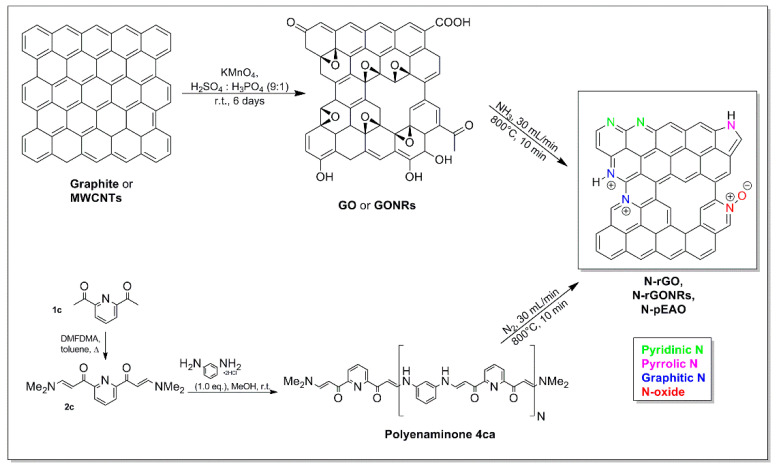
Synthesis of N-rGO, N-rGONRs, and N-pEAO; a schematic reaction scheme.

GO and GONRs possess oxygen functional groups, which were replaced by nitrogen functional groups in N-rGO and N-rGONRs during heat treatment under an NH_3_ atmosphere. On the contrary, N-pEAO preserves the inherent nitrogen functionalities that are part of the synthetic precursors (Scheme 1).

As mentioned above, it is of great importance that, when making a composite membrane, we use graphene-based materials with a high specific surface area and high degree of nitrogen functionalization. These two parameters are responsible for good OH- conductivity and enhancing mechanical membrane properties. Further, structural defects that are formed in the graphene basal plane during the synthesis should also be beneficial for ionic conductivity properties since they make the material permeable for ion diffusion [19]. Another important parameter is the thermal stability of the membrane and its constituents at the operating temperatures of a fuel cell.

**Figure 1:**
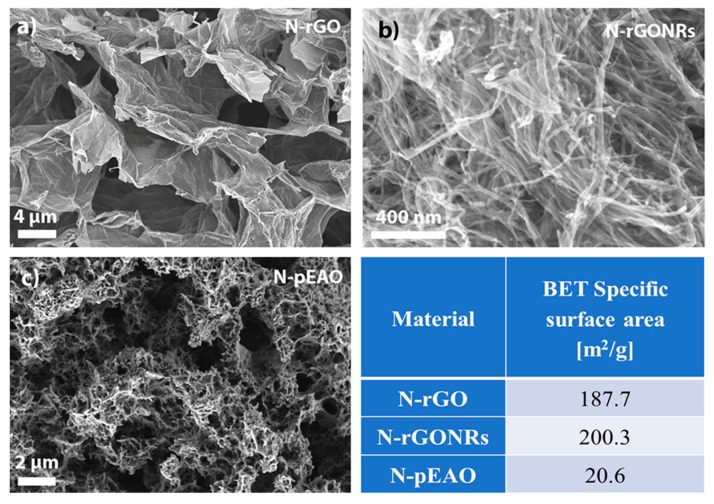
SEM images and Brunauer–Emmett–Teller (BET) specific surface area values of (**a**) N-rGO, (**b**) N-rGONRs, and (**c**) N-pEAO.

N-rGO, N-rGONRs, and N-pEAO were characterized by SEM for morphological purposes. In Figure 1a, the layered nature of the N-rGO is seen clearly. Flakes are several µm in diameter. Further, N-rGO sheets are crumpled, due to freeze-drying of the GO precursor, although a specific surface area of the N-rGO (187.6 m^2^/g) was three times larger compared to the GO precursor (37.2 m^2^/g). It should be noted that specific surface area of N-rGO is measured in a dry form of material, which is known to restack during drying. However, when material is dispersed in liquid media the layers are delaminated, due to the electrostatic repulsion forces, and the specific surface area should increase significantly. N-rGONRs represent a quasi-1D high aspect ratio derivative of a graphene nanoribbon. The SEM image (Figure 1b) shows nanoribbons various sizes clearly, which are twisted, and the edges wrinkled. The BET specific surface area is 200.3 m^2^/g, which was measured again for the freeze-dried sample and is larger than the value for N-rGO. Contrarily, the N-pEAO exhibited a relatively lower BET specific surface area (20.6 m^2^/g), although the SEM morphology (Figure 1c) indicates high porosity—sponge-like morphology.

**Figure 2:**
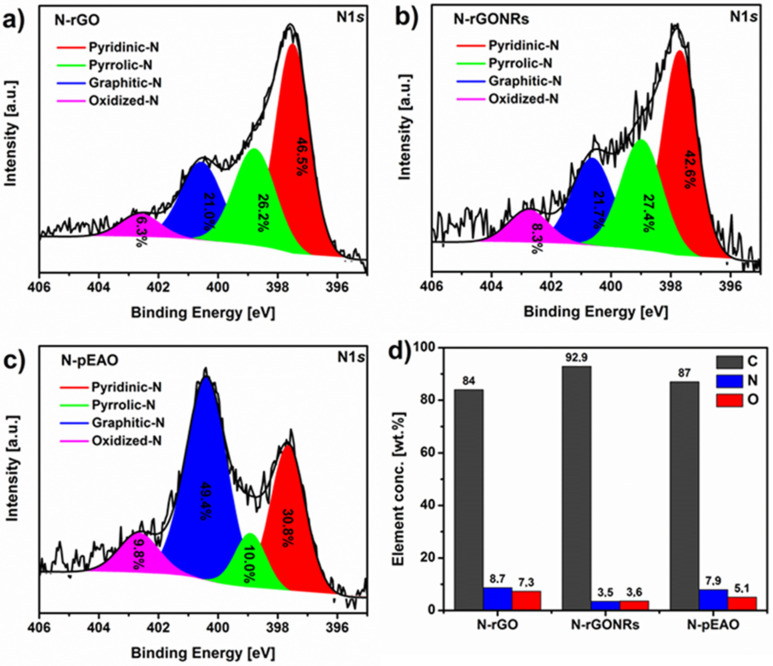
N1s XPS core level spectra with spectral deconvolution and quantified values for (**a**) N-rGO, (**b**) N-rGONRs, and (**c**) N-pEAO, and (**d**) Corresponding quantified values of C, N, and O from the XPS survey spectra.

To evaluate the nitrogen concentration in N-rGO, N-rGONRs, and N-pEAO, we performed CHN elemental analysis (Figure S3) and XPS analysis (Figure 2). Further, we also estimated the concentration of various nitrogen functional groups by deconvolution of the N*1s* core-level spectra. Peaks at 397.5 eV, 398.8 eV, 400.6 eV in 402.7 eV were assigned to the pyridinic N, pyrrolic N, graphitic N, and N-oxide, respectively. Figure 2a–c reveals two different types of materials relative to the nature of nitrogen functionalities. N-rGO and N-rGONRs have a relatively high concentration of pyridinic N (above 42%) and pyrrolic N (above 26%) while N-pEAO has only 30.8% of pyridinic N and 10% of pyrrolic N. Contrarily, the concentration of graphitic N is ~21% for N-rGO and N-rGONRs and 49.5% for N-pEAO. This means that N-rGO and N-rGONRs possess predominantly pyridinic N, while N-pEAO possesses predominately graphitic N. Further, the overall concentration according to the CHN analysis of nitrogen is relatively high for N-rGO and N-pEAO—11 wt.% and 9.8 wt.%, respectively (Figure S3). These values are three times higher than the value for N-rGONRs, which is 3 wt.%. XPS survey values for nitrogen overall concentrations are in correspondence with the CHN analysis. We also noticed that, according to the XPS survey, concentrations of oxygen go up to 7.3 wt.% (Figure 2d). Oxygen is either part of the N-oxide functional group or remaining GO, GONRs, or polyenaminone oxygen functional groups, which might form hydrogen bonds with CS-related functional groups, and, thus, form stable composite membranes.

**Figure 3:**
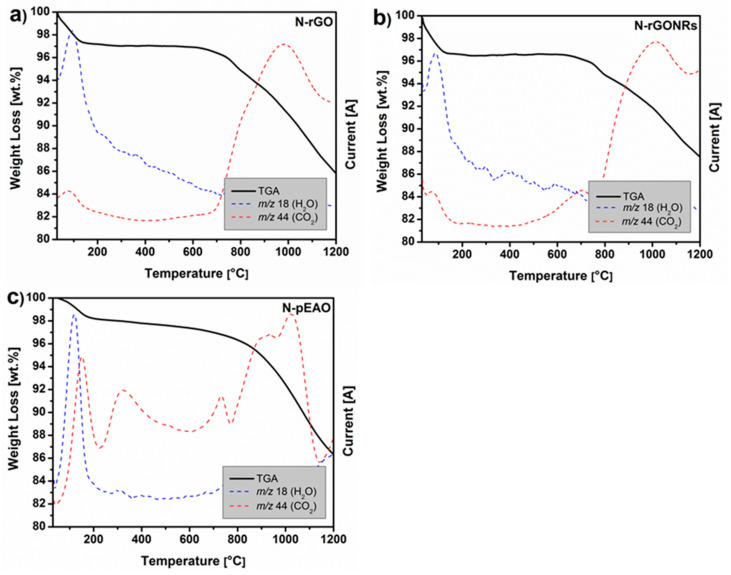
Evolved gas analysis done by TGA (black curve) coupled to MS fragments *m/z* 18 (H_2_O^+^) (blue curve) and *m/z* 44 (CO_2_^+^) (red curve) for (**a**) N-rGO, (**b**) N-rGONRs, and (**c**) N-pEAO.

For testing the thermal stability of N-doped materials, we performed a TGA experiment under dynamic inert conditions (Figure 3). The decomposition gases were also monitored with a mass spectrometer (MS). The TGA results show that N-rGO, N-rGONRs, and N-pEAO are very stable in a wide temperature range, which is beneficial for the stability of the composite membranes during the fuel cell operation conditions. The total weight loss for N-rGO was 14.1 wt.%, for N-rGONRs 12.5 wt.% and for N-pEAO 15.9 wt.%. Further, we observed water uptake from the atmosphere (H_2_O adsorption) for all three materials at low temperature intervals by monitoring the *m/z* 18 signal, which correspond to H_2_O^+^ evolution. Such a property is beneficial for membrane applications since it can lead to the desired electrolyte–membrane interactions. The amount of adsorbed water was 2.6 wt.% for N-rGO, 3.3 wt.% for N-rGONRs and 1.7 wt.% for N-pEAO. Major weight loss was observed (9.1 wt.%, 7.3 wt.%, 10,1 wt.% for N-rGO, N-rGONRs, and N-pEAO, respectively) at very high temperatures, where CO_2_ evolution was in terms of *m/z* 44 was the predominant decomposition gas. This was attributed to the further graphitization and defunctionalization of the remaining oxygen functionalities, which were stable up to 800 °C (maximum pyrolysis temperature in synthesis).

**Figure 4:**
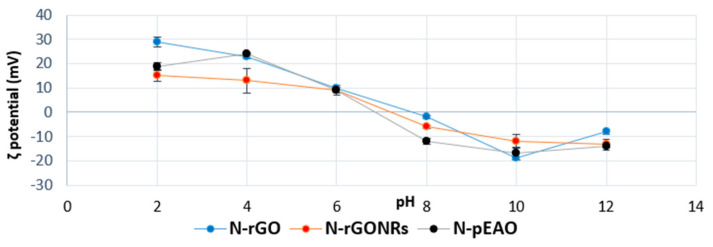
ζ potential of N-rGO, N-rGONRs, and N-pEAO in the pH range 2–12.

N-rGO, N-rGONRs, and N-pEAO water dispersions were examined further by *ζ* potential measurement, which gives an indication of the stability of the colloidal system. The ζ potential curves in Figure 4 demonstrate the pH-switching phenomenon observed for all three dispersions, which can be explained by the Derjaguin–Landau–Verwey–Overbeek theory of colloidal dispersion. According to this theory, colloids tend to agglomerate or remain discrete, according to the net interaction from van der Waals attractions and electrostatic repulsions [20], where the large charge density increases the stability of dispersions by suppressing the aggregation process. The obtained data suggest the largest stability on terminal pH values. Moreover, the relatively low (+10 mV) charge density at pH of mixing with CS solution (pH 6) was measured for N-rGO, N-rGONRs, and N-pEAO particles, which suggests a high probability for aggregation at drying. Having the same net ionization, allow us to exclude this factor when the behavior of differently structured, N-doped graphene derivatives is evaluated in relation to the properties of the final composite membrane.

### 3.2. Chemical Structure, Crystallinity, and Surface Morphology of CS/Graphene-Based Membranes

**Figure 5:**
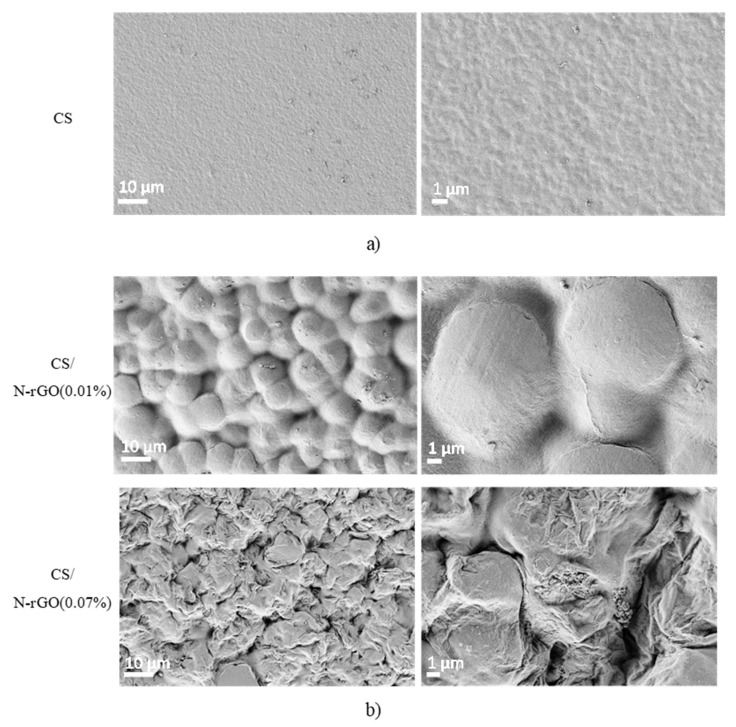

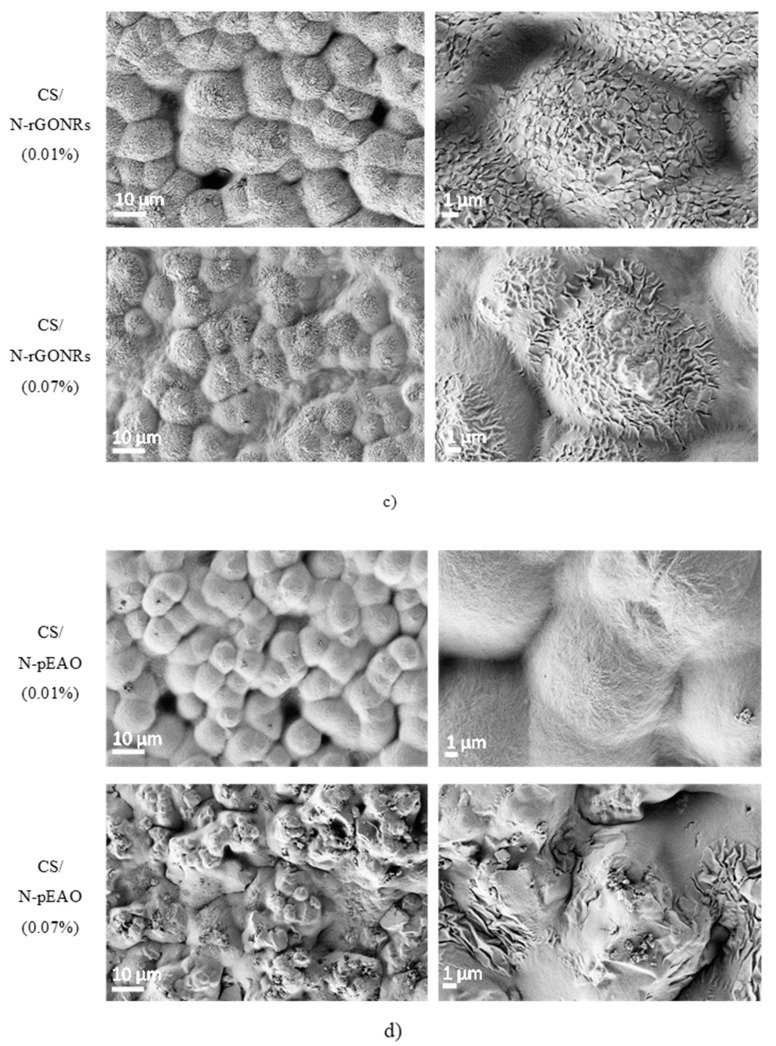
SEM images of (**a**) chitosan (CS) and CS/graphene-based composites, containing 0.01% and 0.07% of (**b**) N-rGO, (**c**) N-rGONRs, and (**d**) N-pEAO.

Figure 5 present the FE-SEM images of CS, CS/N-rGO, CS/N-rGONRs, and CS/N-pEAO membranes, displaying the appearance of membranes’ surface morphology. The structure and morphology of membranes play an important role in ensuring optimal performance of the fuel cells; they influence the conductivity of the system by dictating ion transport, wetting efficiency of the electrolyte, as well as fuel, e.g., ethanol, permeability. Surface morphology further contributes to the operation of the fuel cell, since it can, to a certain degree, even offset the effect of advantageous high conductivity. Our previous study [12] reported that smooth CS membranes pushed the fuel cell performance past samples, which exhibited a more structured and nanoporous surface yet possessed higher conductivity values. The smoothness of the surface was speculated to contribute to a reduction of the interfacial resistance in the membrane electrode assembly, and, thus, resulted in a higher power output.

According to the SEM images (Figure 5), the membranes of CS alone exhibit no discernible structural features (Figure 5a); they possess a non-macroporous, dense film-like structure. CS is well known to form films readily, the physical properties of which are dependent on the concentration of the solution, as well as the type of acid used for the dissolution [21].

The SEM images demonstrate that inclusion of rGO or nitrogen doped graphene-based derivatives into CS polymer matrix cause obvious changes in the morphology of the resulting membranes: The non-structured, homogeneous surface of the polymer film is disturbed to a different degree, depending on the type of graphene-based particle used and its concentration. Morphology of membrane after N-rGO inclusion (Figure 5b) is characterized by a lumpy appearance, with low concentration (0.01%) bulging the CS membrane into tightly packed spherical surface artefacts. These, on a micro-level, retain the smooth surface, as visible in the images of higher magnifications. Increasing the concentration of N-rGO up to 0.07% affects the surface morphology in a pronounced way; while the bulging overall appearance is still discernible (with deeper grooves among the lumps), and on a micro level one can observe the discrete association of graphene-based particles within the CS matrix. This can be attributed to an increase in concentration from 0.01% to 0.07% of particles, and despite that, one can regard the higher value as a still relatively low concentration value for particle inclusion, which does result in limited phase separation. Nonetheless, it must be noted that graphene-based particles are still well embedded and well dispersed within the CS polymer phase.

The membrane set produced with N-rGONRs inclusion into CS (Figure 5c), again exhibited the bulging of the carrier CS membrane, where the ribbon-shaped particles obviously bring about specific structural features on a micro-level. In the case of the lower concentration (0.01%), these appear to adopt a flat conformation within the CS polymer membrane, covering the entire surface of the membrane evenly by stacking laterally next to each other, and affording the sample with pores along the sides of the contacting particles. Upon the increase of concentration (up to 0.07%) N-rGONRs particles assume a different arrangement, again stemming from the association of particles, as suggested by the ζ potential data. Particles do not appear in a flat fashion, but rather create a creased film structure, localized on the elevated portions of the membrane surface. Opposite to the previous samples, the CS membranes including N-pEAO particles (Figure 5d) exhibit, regardless of the concentration, a less ordered surface in terms of the bulging effect of the polymer matrix; here, much deeper voids and holes are present. On a micro-level, lower concentration yields a surface, which exhibits a fibrillar-like appearance with particles causing shallow homogeneous ripples within the polymer film. A concentration value of 0.07% alters the surface morphology drastically, where unevenly shaped and sized lumps appear with randomly scattered agglomerates of N-pEAO particles within the polymer matrix, which is a consequence of their 3D porous shape. On the contrary, the 2D N-rGO and quasi-1D N-rGONRs-containing membranes exhibit a homogeneous appearance with evenly distributed structural features encompassing large areas, which reflects the capacity of CS to provide an efficient embedding medium for particle inclusion, as well as performing the function of a supporting substrate on a larger scale, diminishing the possibilities that membranes could fail due to localized inhomogeneity.

**Figure 6:**
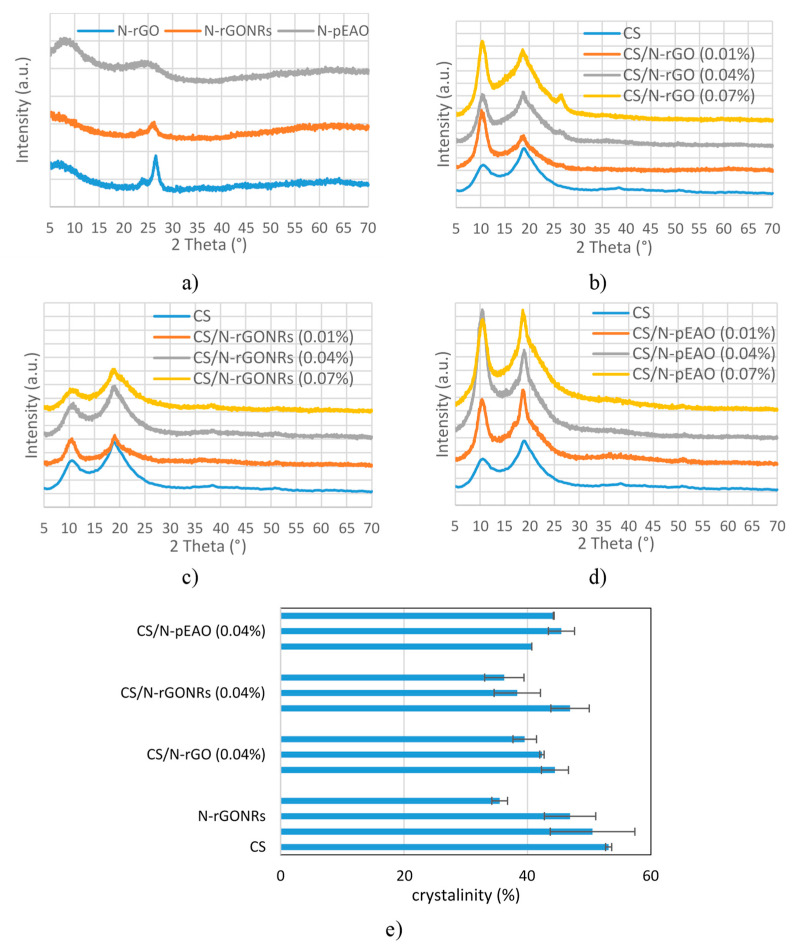
XRD patterns of (**a**) N-rGO, N-GONRs and N-pEAO and CS/graphene composites, containing 0.01% to 0.07% of (**b**) N-rGO, (**c**) N-rGONRs, (**d**) N-pEAO, and (**e**) calculated crystallinity index (CrI_020_ = (*I*_020_ − *I*_am_)/*I*_020_ × 100 and CrI_110_ = (*I*_110_ − *I*_am_)/*I*_110_ × 100), where *I*_110_ represents the maximum absorption intensity during 18–20°, and *I*_110_ represents 11° [23]. The CS spectrum was added as control (**b**–**d**).

XRD was used to identify the crystallinity profile of CS-graphene-based membranes, in particular the effect of the different graphene component on it. The XRD pattern of N-rGO and N-rGONRs (Figure 6a) demonstrate a diffraction peak at the vicinity of 26°, corresponding to an (002) plane typical for thermally reduced GO [22], where there is a small difference in % crystallinity (only ~5% lower in N-rGONRs compared to N-rGO with~50% crystallinity). The N-pEAOs have broad diffraction in the vicinity of 25°, demonstrating more turbostratic structure as a consequence of the parent compound structure (amorphous, 3D porous) [18].

The molecular organization of CS chains within the membranes is influenced strongly by the processing procedure, type of acids and alkaline solutions used in dissolution and neutralization, respectively, as well as its molecular characteristics, such as the Mw, degree of deacetylation, origin, etc. [24]. The crystallinity of CS is determined by the intramolecular and intermolecular hydrogen bonds of its tertiary structure. The XRD pattern of neat CS membrane (Figure 6a) demonstrates two dominant reflections at 2*θ* = 11° (020) and 2*θ* = 18° (110), which coincide with the pattern of the form I crystal and the form II crystal, respectively. This membrane also has the highest (mean) crystallinity index (53.2%). In all cases, the addition of graphene-based components reduces the crystallinity. The increase of N-rGO and N-rGONRs concentration (up to 0.07%) reduce the crystallinity down to a value of 39.55% and 36.25%, respectively. This means that crystallization of CS in affected negatively by the presence of N-doped rGO components, which is opposite to the recent findings for CS/GO aerogels [23], due mainly to the use of N-doped rGO and not GO itself. In the case of N-pEAO addition, the order of crystallinity change is opposite, and the same was not influenced significantly by increasing concentration, which we speculate to be due to the 3D macroporosity of N-pEAO (Figure 1c), allowing migration of CS within its structure and closer interaction. The latter can be supported by the high magnification SEM images (Figure 5d), revealing smooth (amorphous) film formation at lower and more ordered aggregations at higher loading.

**Figure 7:**
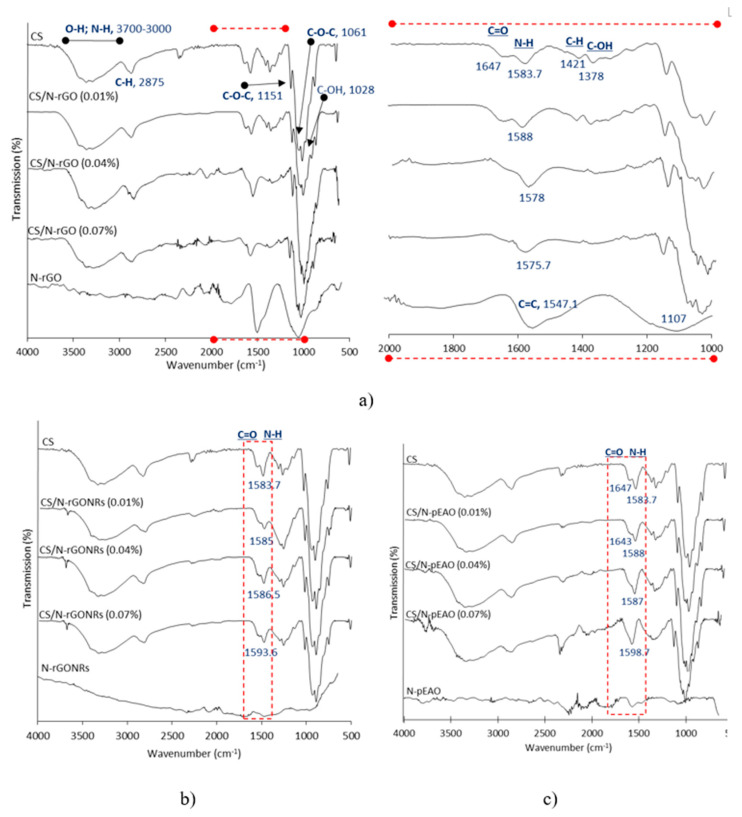

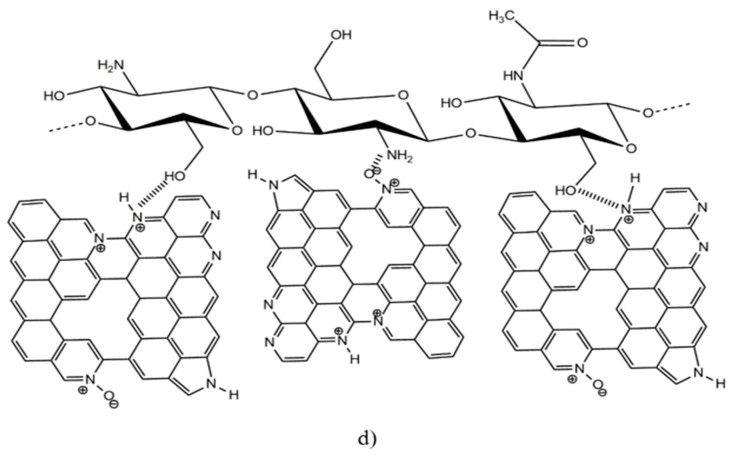
FTIR spectrum of neat CS and CS/graphene composite membranes, containing 0.01%, 0.04% and 0.07% of (**a**) N-rGO, (**b**) N-rGONRs and (**c**) N-pEAO. The spectra are offset vertically to aid visibility. (**d**) Tentative scheme of CS-graphene interaction within composite membranes.

ATR-FTIR analysis aims to identify the potential interactions amongst membrane components, the CS and N-doped graphene components. The vibration spectra of the CS membrane (Figure 7, top-most spectral line on each graph) evidenced typical CS polymer bands, assigned to O-H and NH_2_ vibrations (3700–3000 cm^−1^), C-H stretching vibration (3000–2800 cm^−1^), C=O stretching, N-H bending vibrations within the residual amide N-acetyl (NHCOCH_3_) group and CH_2_ bending vibration in the CH_2_OH group (1647 cm^−1^, 1583.7 cm^−1^ and 1421 cm^−1^, respectively), C-OH stretching of the primary alcohol group (1378 cm^−1^), symmetric and asymmetric stretch of a C-O-C glucoside bridge (~1151 cm^−1^ and ~1061 cm^−1^, respectively) and C-O vibration of a secondary OH group (~1028cm^−1^) [25].

Figure 7 demonstrates the FTIR spectrum of the CS composite membranes, containing different concentrations of graphene-based particles N-rGO (a), N-rGONRs (b), and N-pEAO (c), where the spectrum (a) on the right is the detailed figure which focused on the spectral range of interest (~1600 cm^−1^ to 1400 cm^−1^, covering the N-H and C=O vibration regions), which is most affected by components’ mixing. The absorption band at 1647 cm^−1^ is characteristic of the C=O stretching mode of the amide group, while 1583.7 cm^−1^ is related to the bending mode of the N-H in the primary amine. The position of both is affected by addition of N-doped graphene particles within the CS polymer matrix, as observable vibration shifts (respective to the CS band position, as control) are present: the N-H vibration band shifts from 1583.7 cm^−1^ (in CS) to higher wavenumbers, i.e., 1588 cm^−1^, 1585 cm^−1^ and 1588 cm^−1^ in CS/N-rGO, CS/N-rGONRs, and CS/N-pEAO, respectively, in the case of the lowest (0.01%) loading. Increasing the loading percentage (up to 0.07%), further shifted the vibration to higher 1593.6 cm^−1^ and 1598.7 cm^−1^ in CS/N-rGONRs and CS/N-pEAO, respectively, while opposite shifting (to 1575.7 cm^−1^) was observed in the case of CS/N-rGO, the latter due to interference with the C=C related band at 1547 cm^−1^. These shifts indicate hydrogen bonding between CS amines and the rGO (remaining) oxide groups (tentative scheme in Figure 7d), which were also detected by XPS. To this end, the covalent bonding between both components, as described in the literature for a similar CS/rGO system [26], can hardly be speculated here, as no increase in intensity of vibration at 1647 cm^−1^ (C=O band), relative to the intensity of band related to N-H (1583.7 cm^−1^) can be detected as confirmation of new amide bonds among the residual oxygen groups of N-doped graphene components and CS amines [27]. To sum up, shortly, the largest change was observed within the CS/N-pEAO composite membranes, where significant reduction in intensity down to complete disappearance was observed for the 1647 cm^−1^ band, with concentration increase of the N-pEAO component, which aligns with the XRD findings of the largest crystallinity, potentially due to the largest interfacial contact and bonding between both components. Importantly, the polyenaminone oxygen functional groups, remaining also in the N-pEAO product (9.79% of N-O groups were detected by the XPS survey and CHN elemental analysis, Figure 2c), and the same can form hydrogen bonds with the CS-related NH_2_ or NHCOCH_3_ group, which can be responsible for the described larger changes in the amide region for this sample.

### 3.3. Mechanical Properties

As mentioned in the beginning, the rationality behind the addition of N-rGO, N-rGONRs, and N-pEAO to the CS matrix is not only to enhance the ionic conductivity, but also to reinforce the relatively weak CS membrane mechanically.

**Figure 8:**
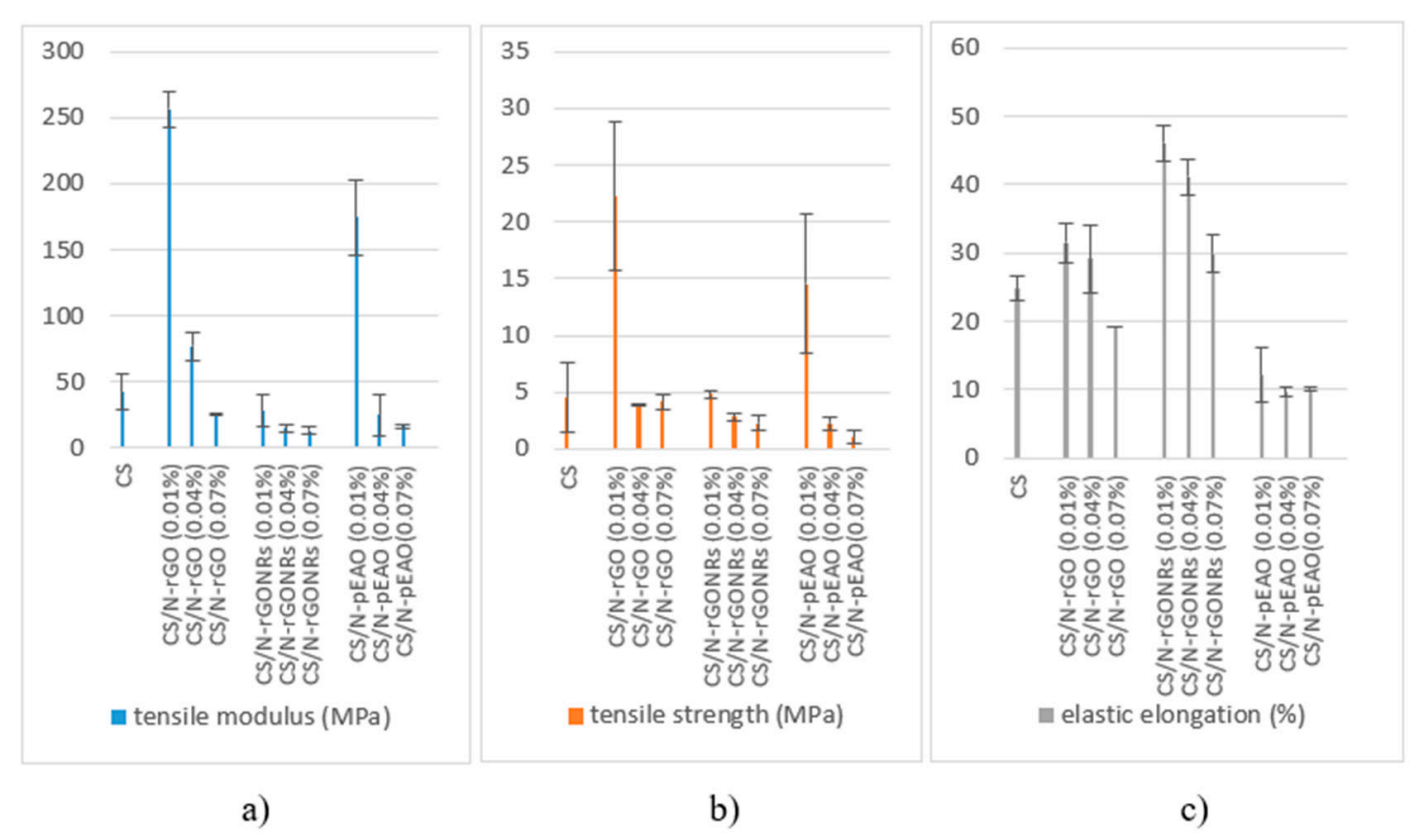
(**a**) Tensile modulus (MPa), (**b**) Tensile strength (MPa) and (**c**) Elastic elongation (%) data obtained at tensile testing of neat CS and CS/graphene composite membranes, containing 0.01%, 0.04%, and 0.07% of N-rGO, N-rGONRs and N-pEAO.

Results obtained by the tensile test (Figure 8) demonstrate that the neat CS membrane has a tensile modulus of 42.26 ± 13.5 MPa, tensile strength of 4.52 ± 3.1 MPa and elastic elongation of 24.73 ± 1.8%. Separate addition of low concentration (0.01%) N-rGO and N-pEAO improved the tensile modulus (506.8% and 312.7%, respectively) and tensile strength (392.5% and 220.9%, respectively) significantly, which can be attributed to their structural features (2D and 3D, versus 1D shape) and orientation within the CS matrix, rather than superior dispersibility over the N-rGONRs, as the same ζ potential (Figure 1) of ~10mV was measured at pH 6 for all types. We assume that the in-plane direction of performing the tensile strength affects the results in the way that a sheet-like orientation of the filler (as typical for flake-like rGO) is most beneficial. Reduction of tensile modulus and tensile strength in the case of N-rGONRs’ addition can be attributed to the disturbance of the interaction between CS polymers upon the addition of this component [12], already at low concentration, where even the largest surface area of 200.3 m^2^/g (BET data) compared to other fillers cannot compensate. Addition of higher concentrations of each type of filler reduce the tensile modulus and tensile strength values, which are expected and reported phenomena when graphene-derivatives are dispersed within the CS films, instead of reduction after membranes’ formation; in the latter, the short-range interactions are more intensive, due to the proximity of both components [28]. Previous studies [29] utilizing CS and GO as membrane components explained that, at low content, GO can be dispersed in the polymer solution uniformly and had good adhesion with the polymer matrix, while, with the further increase of the GO content, the GO dispersion in the polymer solution deteriorated, resulting in poorer adhesion with the polymer and, thus, decreased the mechanical property of the membrane. The strengthening effects of GO on polymers is related mainly to the properties of GO nanolayers, the dispersion state of GO in the polymer, the interphase interactions, as well as changes in the crystalline structures of semi-crystalline polymers. As is obvious, the lower contents, e.g., 0.01%, lead to better dispersion and, therefore, more efficient reinforcement.

High magnification SEM images reveal continuous, film-like features in the case of the CS membrane with 0.01% N-rGO and N-pEAO, while discontinuous, the cracked surface was seen in case of N-rGONRs, which we anticipate having a negative effect on mechanical properties. On the contrary, the elastic elongation was highest for N-rGONRs containing CS membranes and lowest for N-pEAO containing CS membranes at respective concentrations, which implied elasticity introduced by the ribbons-shaped rGO, while sheet-like N-rGO and porous N-pEAO were relatively more rigid within the (in-plane) direction, which was adapted during the tensile test.

### 3.4. KOH Uptake, in Plane Swelling and Ethanol Permeability

For a given AEM, the KOH uptake of the polymeric membrane is a critical parameter, because if it goes too high it may lead to cathode flooding, whereas going too low may result in poor transport properties, lowering the cell performance. Moreover, the KOH uptake has a significant effect on OH- ion conductivity and the overall properties of the membrane, especially in the case of highly hydrophilic polymers, such as CS.

**Figure 9:**
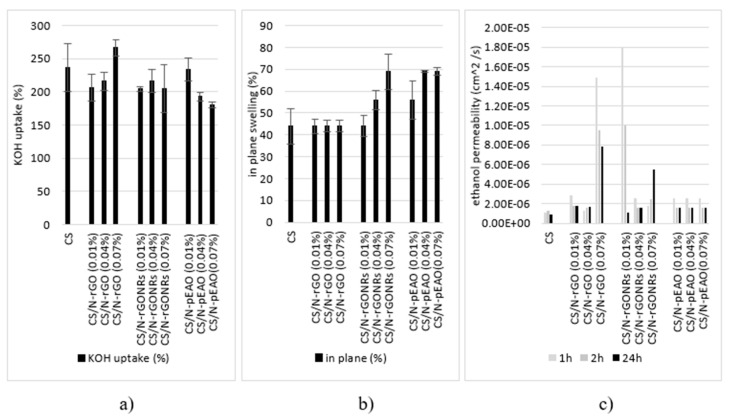
(**a**) KOH uptake (%) and (**b**) in-plane swelling (%) data, obtained after 24 h immersion in 6M KOH, and (**c**) ethanol permeability (cm^2^/s), obtained after 1 h, 2 h, and 24 h measurement within a constructed cell of neat CS and CS/graphene composite membranes, containing 0.01%, 0.04%, and 0.07% of N-rGO, N-rGONRs and N-pEAO, all estimated as a working temperature of 25 °C.

Figure 9 demonstrates the (a) KOH uptake and (b) Swelling ratio of 1% CS membranes with the addition of a N-rGO, N-rGONRs and N-pEAO at three different concentrations (0.01%, 0.04%, and 0.07%).

A neat CS membrane has a relatively low KOH uptake of 236 ± 35.7%, compared to CS swelling in water, due mainly to deionization at such high pH [30], which is expected for a non-crosslinked membrane made from highly hydrophilic biopolymer. The KOH uptake was reduced by 18.2% and 23.5% after loading with 0.04% and 0.07% N-pEAO, respectively (Figure 9a). These membranes demonstrate the most pronounced interactions between the components (as seen by FTIR), and high magnification SEM visualized an integrated system, which influenced the mechanical properties positively, and restricted large KOH uptake. The same membranes demonstrated filler concentration-dependent in-plane swelling, which may be a consequence of the adaption of CS molecules around/within the 3D porous N-pEAO particles, and we speculated that a non-restricted chain relaxation occurred due to molecular sorption in 6 M KOH. In the case of CS loading with the largest (0.07%) concentration of N-rGO, the KOH uptake increased by 12.8%, which was the largest increase among all membranes containing filler. The same sample demonstrated visible phase separation between CS and N-rGO on the high magnification SEM, and will be described in the next paragraph as the membrane with the highest ethanol permeability. Nevertheless, the same sample did not evidence the larger in- plane swelling compared to other concentrations of the same filler, which implied possible through plane swelling due to the specific orientation of CS/N-rGO within the membrane, where a not well-adhered interphase allows larger KOH intake. Swelling to a certain extent is useful, as the membrane maintained better contact with the gas diffusion electrode and minimized the interfacial resistance of the cells during hydration/dehydration cycles [31].

Fuel permeability is one of the key parameters that must be considered in evaluation of membrane performance. In the case of ethanol permeability through the membrane, a reduction occurs in the electrolytic activity of cathode-catalytic catalysis, density, and fuel consumption in fuel cells. Ethanol mixes instantly with water, and, thus, penetrates the OH- conducting membrane easily, where it can combine and react with the migrated electrocatalysts to accelerate the degradation process. On another hand, ethanol can pass through the membrane, entering the cathode and interfering with the cathode reaction, in addition to electrocatalyst poisoning.

Ethanol permeability measurement was carried out through an ex-situ test, using the diffusion model. A cell consists of two glass reservoirs: reservoir A filled with 25 mL of 6 M KOH and reservoir B filled with 25 mL of 2 M EtOH/6 M KOH. The membrane, with an area of 66.04 cm^2^, is located between the two temperature-controlled (thermostated at 25 °C) diffusion cells. The ethanol concentration was determined indirectly, using calibration curve plotting conductivity of different EtOH/6M KOH mixtures. According to the results in Figure 9c, the lowest ethanol permeability after 1 h exposure in the cell was measured for neat CS (reference), and was 9.25 × 10^−7^ cm^2^/s, which is comparable to the literature reports using crosslinked CS under higher temperature (40 °C) [32]. The permeability was found to increase after the addition of filler in all three cases, and the most pronounced (close to 10-fold) increase was observed in CS/N-rGO (0.07%) and CS/N-rGONRs (0.01%) membranes. We anticipate that the presence of fillers, especially in cases when low interfacial adhesion between components is present, induces formation of intermediate spaces, which do not restrict the migration of ethanol molecules. In the case of CS/N-rGONRs (0.01%), such voids were even visible on a large-scale, appearing as surface cracks on high magnification SEM micrographs. A less affected composite membrane, with the lowest increase of ethanol permeability relative to neat CS, was observed in membranes containing N-pEAO, which we assumed to be a consequence of the 3D porosity of the filler material, allowing inclusion of CS molecules, close packing, and the already described molecular (H-bonding) interactions. This, in turn, reduced the permeability; the same filler demonstrated a reinforcing effect, also improving the mechanical properties. The relation of mechanical properties and ethanol permeability also aligned well in the case of the sample with the highest permeability, i.e., CS/N-rGONRs (0.01%), the same having the lowest tensile strength and modulus, which was attributed to its (1D) structural organization. This membrane was also the only sample where increase of filler concentration reduced the permeability, which was not seen in N-pEAO and N-rGO, the latter even increasing the permeability. Such random behavior can only be explained by the function of the filler shape and its orientation, guiding the structuring of the composite membrane as whole.

At 24 h measurement, clear relations among composites were observed, in terms of ethanol permeability, where increasing of filler concentration (from 0.01% to 0.07%) increased the permeability, excluding the membrane containing N-pEAO, where similar values were obtained for all concentrations. Moreover, in almost all cases, permeability was reduced with time, which may be related to swelling of the membrane and/or migration of fillers, which can only be speculation at this point. We expect that, at one follow up study, the introduction of a zero or short length crosslinker, can evoke closer packaging, and improve the ethanol permeability profile, simultaneously improving the mechanical profile as well.

### 3.5. Performance of CS-Based Membranes within a Single Cell Test

**Figure 10:**
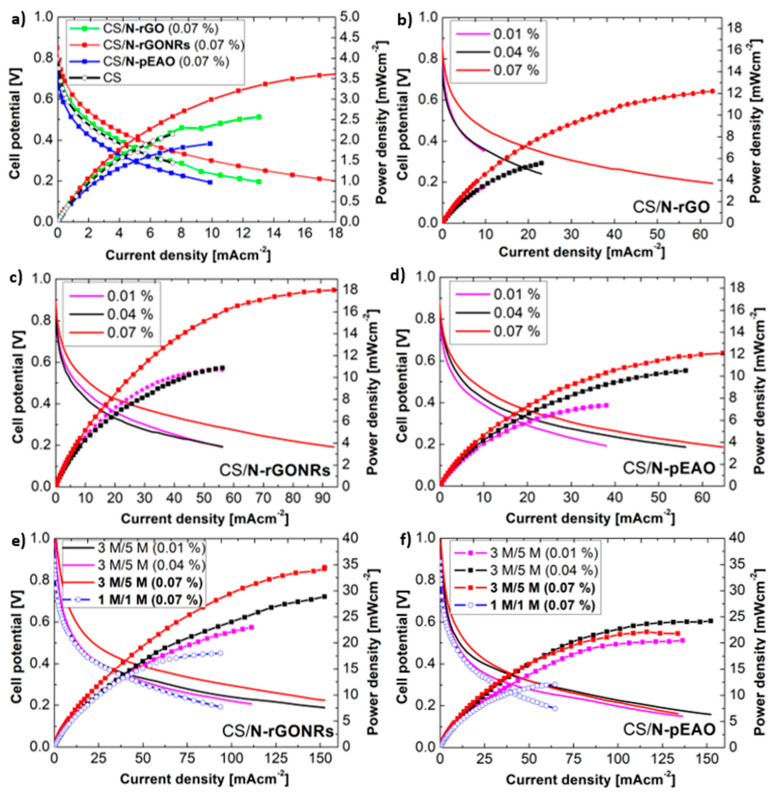
Typical Direct Ethanol Alkaline Fuel Cell (DEAFC) discharged cell voltage and power density of (**a**) CS membranes at room temperature with 1 M EtOH/1 M KOH, (**b**–**d**) CS membranes at 57 °C and 1 M EtOH/1 M KOH, and (**e**–**f**) CS membranes at 57 °C and 3 M EtOH/5 M KOH.

The membrane performance in fuel cells is determined in direct alkaline ethanol test cells. The effect of different N-doped graphene-based components and their loading concentrations was investigated, as well as the influence of temperature and fuel concentration on the DAEFC membrane performance. Figure 10 shows typical DAEFC I V (left axis) and I-P (right axis) plots of the pristine CS membrane, as well as of the as prepared CS/N-rGO, CS/N-rGONRs, and CS/N pEAO membranes. The cell voltage and the maximum power density of all samples increased with ascending temperatures, independent of the fuel concentration (Figure S4, Table 1). CS/N-rGONRs (0.07%) shows as an example an increase in open circuit voltage (OCV) from 737 to 895 mV, and maximum power densities of 3.6–10 mWcm^−2^ at a fuel mixture of 1 M EtOH and 1 M KOH when the cell temperature was raised from ambient temperature to 57 °C. In a fuel solution of 3 M EtOH/5 M KOH at 57 °C, the OCV was approx. 150 mV and P_max_. approx. 13 mWcm^−2^ higher than at ambient temperature. This is consistent with similar studies [31] and is attributed to the improved reaction kinetics at the electrodes and conductivity of the cell at higher temperatures.

Measurements with higher concentrated fuel were carried out for the CS/N-rGONRs and CS/N pEAO to test the influence of EtOH and KOH concentration on membrane performance in test cells. The cell voltage and the maximum power density increased when a mixture of 3 M EtOH/5 M KOH was used instead of 1 M EtOH and 1 M KOH, as can be seen in Figure 10e,f. CS/N-rGONRs (0.07%) had a 17 mVcm^−^^2^ higher P_max_. and the OCV was 200 mV higher for the concentrated fuel. OCV and P_max_. of CS/N pEAO (0.07%) increased from 827 mV and 12 mWcm^−^^2^ to 1017 mV and 22 mWcm^−^^2^. The higher cell performance, especially in the low current density region, was caused on the one hand by the fact that the ethanol availability was improved, thus ensuring sufficient ethanol adsorption and OH^−^ on the active surface. Therefore, the reaction kinetics in the EOR were enhanced and led to better cell performance. On the other hand, the cell performance was affected by the higher alkaline anode feed concentration. The OH^−^ ion conductivity of the AEM contributes mainly to an increase of cell voltage and power density. In addition, more OH^−^ ions are provided for faster EOR kinetics and mass transport at the anode [33].

The influence of the addition of N-doped graphene-based components to the CS membranes on the Direct Ethanol Alkaline Fuel Cell (DEAFC) performance was examined by comparing CS/N-rGO, CS/N rGONRs and CS/N pEAO with the pristine CS membrane. In Figure 10a, the I-V and I-P plots of the CS membranes containing the highest N-rGO, N-rGONRs and N-pEAO concentration (0.07%) are compared with blank CS membrane at room temperature and 1 M EtOH/1 M KOH anode fuel. CS/N rGONRs (0.07%) significantly (P_max_. = 3.7 mWcm^−2^) and CS/N-rGO (0.07%) slightly (P_max_. = 2.5 mWcm^−2^) outperformed the performance of the pristine CS membrane (P_max_. = 2.2 mWcm^−2^), whereas CS/N pEAO (0.07%) illustrated lower cell potential and power density values. This shift in power output might be explained by the surface morphology, mechanical properties, and conductivity of the membranes. A smooth surface reduces the interfacial resistance in the membrane electrode assembly [12]. Therefore, pristine CS might show better cell performance at room temperature than CS/N pEAO. The smoother surface morphology of CS/N-rGO and CS/N rGONRs compared to CS/N pEAO was compensated by a higher membrane conductivity, resulting in a better performance of the blank CS membrane. In addition, elastic elongation was the highest for N rGONRs containing CS membranes, and the lowest for N-pEAO, which also influenced the quality of the MEA.

In contrast to lower temperatures, CS/N pEAO exceeded the performance of CS/N-rGO at higher temperatures, whereas CS/N rGONRs still showed the highest values (Figure 10e,f, and Table S1). In a 3 M EtOH/5 M KOH solution, CS/N rGONRs also showed higher maximum power densities compared to the CS/N pEAO samples. The reason for this might be changes in the quality of the MEA at higher temperatures. In addition, N rGONRs had the highest specific surface area according to the BET measurements of 200 m²/g, possessing predominantly pyridinic N (above 42 at.%) instead of graphitic N, and presented the highest water uptake from the atmosphere.

The effect of N-doped graphene-based loading concentrations in the 1 M EtOH/1 M KOH anode fuel is illustrated in Figure 10b–d. The cell performance increased for all three samples with ascending N-doped graphene-based loading. CS/N-rGO showed the highest increase of P_max_. from 3.4 mWcm^−2^ to 12 mWcm^−2^ when the N-rGO was raised from 0.01% to 0.07%. The maximum power density of CS/N rGONRs and CS/N pEAO was 7 mWcm^−2^ and 4.5 mWcm^−2^ higher, respectively. This correlated with the different mechanical properties at a higher N-doped graphene-based loading, lower crystallinity, and a higher swelling ratio. Swelling to a certain extent is useful in improving contact with the gas diffusion electrode and minimizing cell interfacial resistance during hydration/dehydration cycles [31].

In summary, the CS/N rGONRs with the highest N-doped graphene-based loading (0.07%) at a temperature of 57 °C and the concentrated 3 M EtOH/5 M KOH anode fuel presented the best overall performance. The OCV of 1095 mV was close to the theoretical voltage of 1140 mV, and a P_max_. of 34.5 mWcm^−2^ could be achieved at a current density of 152.54 mAcm^−2^. In previous studies [19], the highest reported power density value for CS-based membranes was 62.2 mWcm^−2^ (current density 174 mAcm^−2^) at 60 °C and 72.7 mWcm^−2^ (current density 209 mAcm^−2^) at 80 °C. Compared to these investigations, lower temperatures (57 °C instead of 60 °C and 80 °C), a lower oxygen fuel rate (25 mLmin^−1^ instead of 100 mLmin^−1^) and lower metal loading for the anode (0.75 mgcm^−2^ instead of 2 mgcm^−2^) and for the cathode (0.5 mgcm^−2^ instead of 1 mgcm^−2^) were used. Despite these changes or reductions, excellent results were achieved.

## 4. Conclusions

Use of anion exchange membranes as ion-conducting polyelectrolytes within fuel cells is a promising, cost-effective approach, where research is focused on their stability, permeability, conductivity, and ultimate cell performance. We have shown that N-doped graphene derivatives (N-rGO, N-rGONRs, and N-pEAO) play an important role in CS-based nanocomposite membranes, which are relevant for direct alkaline ethanol fuel cells. In particular, N-doped graphene derivatives in concentrations between 0.01 wt.% and 0.07 wt.% change the morphology of the CS membrane drastically due to interactions between the functional groups of CS and N-doped graphene derivatives, as well as (1D, 2D, and 3D) shape of latter. In addition, properties, such as crystallinity, tensile modulus, tensile strength, KOH uptake, and ethanol permeability within composite membranes were also changed compared to pure CS membranes. The synergistic contribution of graphene derivatives and CS in membranes were tested finally in direct alkaline ethanol test cells. We demonstrated that nanocomposite membranes containing N-rGONRs exceeded the fuel cell performance of the pristine CS membrane significantly. Encouraging fuel cell results point to new sustainable, bio-based membranes for use in alternative energy conversion device applications.

## Figures and Tables

**Table 1:** Open circuit voltage (OCV) and P_max_. of CS/N-rGONRs (0.07%) as a function of temperature and concentration.

Temperature	OCV (mV)	P_max._ (mWcm^−2^)	OCV (mV)	P_max._ (mWcm^−2^)
	1 M EtOH/1 M KOH		3 M EtOH/5 M KOH	
RT (22 °C)	789	3.6	948	11.9
35 °C	815	6.7	1003	20.4
43 °C	848	10.1	1039	26.3
50 °C	869	13.8	1082	33.6
57 °C	895	18.0	1095	34.5

## Data Availability

The data presented in this study are available on request from the corresponding author. The data are not publicly available due to privacy reasons.

## Supplementary Materials

The following are available online at https://www.mdpi.com/1422-0067/22/4/1740/s1. Figure S1. SEM image of Polyenaminone 4ca [13]. Figure S2. Raman spectra of (a) N-rGO, (b) N-rGONRs, and (c) N-pEAO. Figure S3. CHN elemental analysis of N-rGO, N-rGONRs, and N-pEAO. Figure S4. Typical DEAFC discharged cell voltage and power density of CS/N-rGONRs (0.07%) as a function of temperature in (a) 1 M EtOH/1 M KOH and (b) 3M EtOH/5M KOH. Table S1. Pmax. and related current density of CS membranes at 57 °C.
