# Supplementary material for: Efficient Chitosan/Nitrogen-Doped Reduced Graphene Oxide Composite Membranes for Direct Alkaline Ethanol Fuel Cells
Source: Int J Mol Sci. 2021 Feb 9;22(4):1740. doi: 10.3390/ijms22041740 (PMC7916145; doi:10.3390/ijms22041740)
Supplement: Supplementary file 1 [file ijms-22-01740-s001.pdf]

SUPPORTING INFORMATION

Efficient Chitosan/Nitrogen-doped Reduced Graphene Oxide Composite Membranes for Direct Alkaline Ethanol Fuel Cells

Selestina Gorgieva[1,2], Azra Osmić[2], Silvo Hribernik[1,2], Mojca Božič[3], Jurij Svete[5], Viktor Hacker[4], Sigrid Wolf[4] and Boštjan Genorio[5]*

[1]Faculty of Mechanical Engineering, University of Maribor, Smetanova 17, 2000 Maribor, Slovenia

[2]Faculty of Electrical Engineering and Computer Science, University of Maribor, Koroška cesta 46, 2000 Maribor, Slovenia

[3]Dravske elektrarne Maribor d. o. o., Obrežna ulica 170, 2000 Maribor, Slovenia

[4]Graz University of Technology, Institute of Chemical Engineering and Environmental Technology, Stremayrgasse 9, 8010 Graz, Austria

[5]University of Ljubljana, Faculty of Chemistry and Chemical Technology, Večna pot 113, SI-1000 Ljubljana, Slovenia

Corresponding author

Assist. Prof. Dr. Bostjan Genorio

University of Ljubljana, Faculty of Chemistry and Chemical Technology

Večna pot 113, SI-1000 Ljubljana, Slovenia

Tel.: +386 1 479 8586

E-mail: bostjan.genorio@fkkt.uni-lj.si

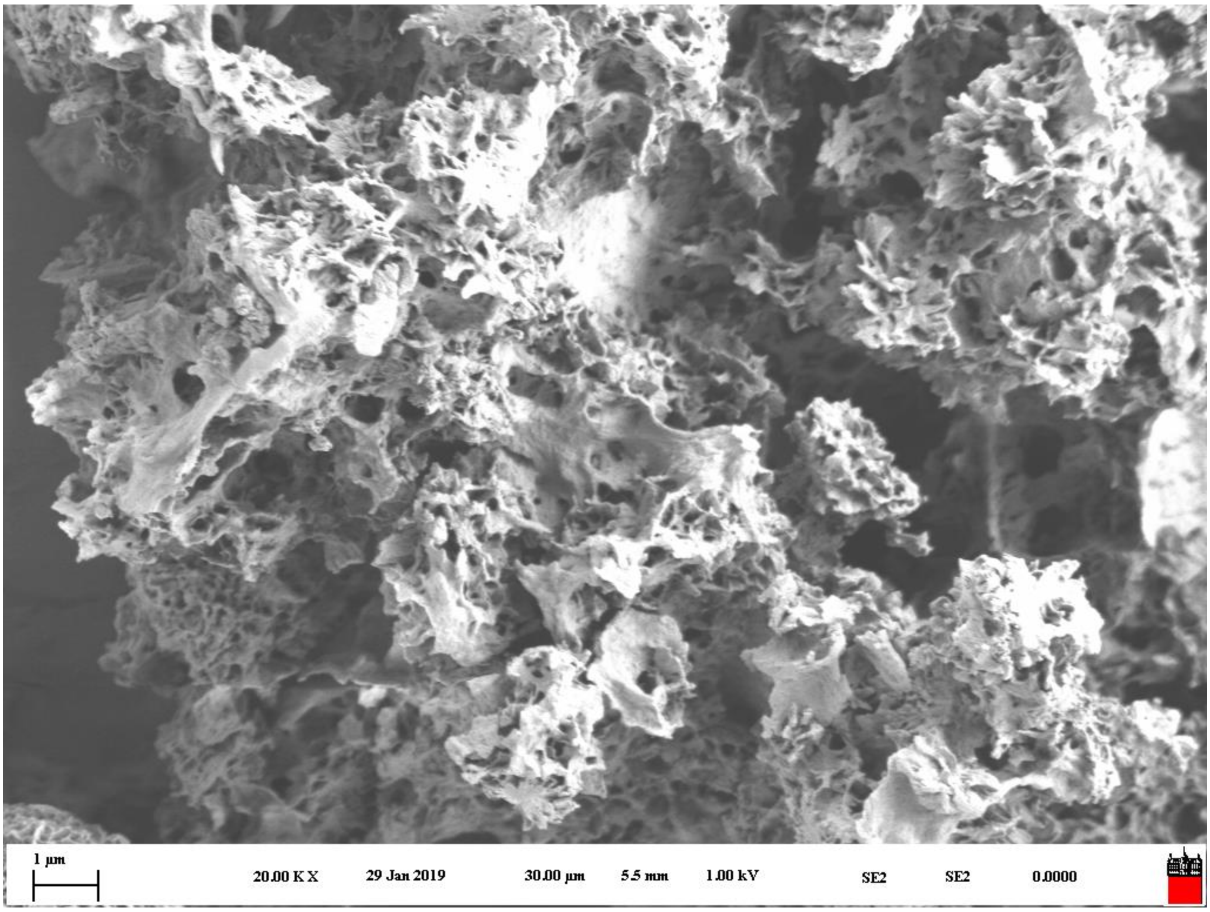

Figure S1. SEM image of **Polyenaminone 4ca[13].**

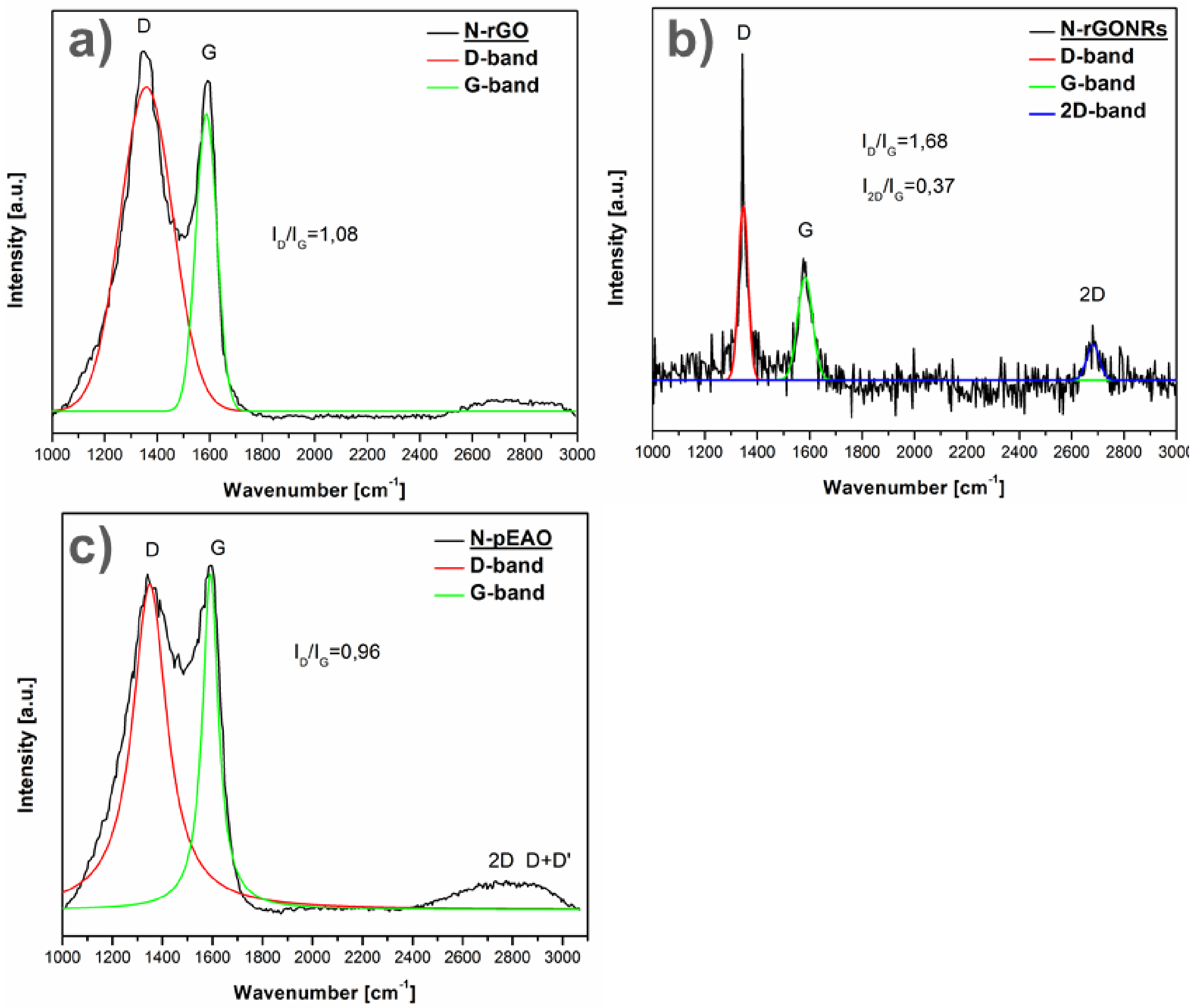

Figure S2. Raman spectra of a) **N-rGO**, b) **N-rGONRs**, and c) **N-pEAO**.

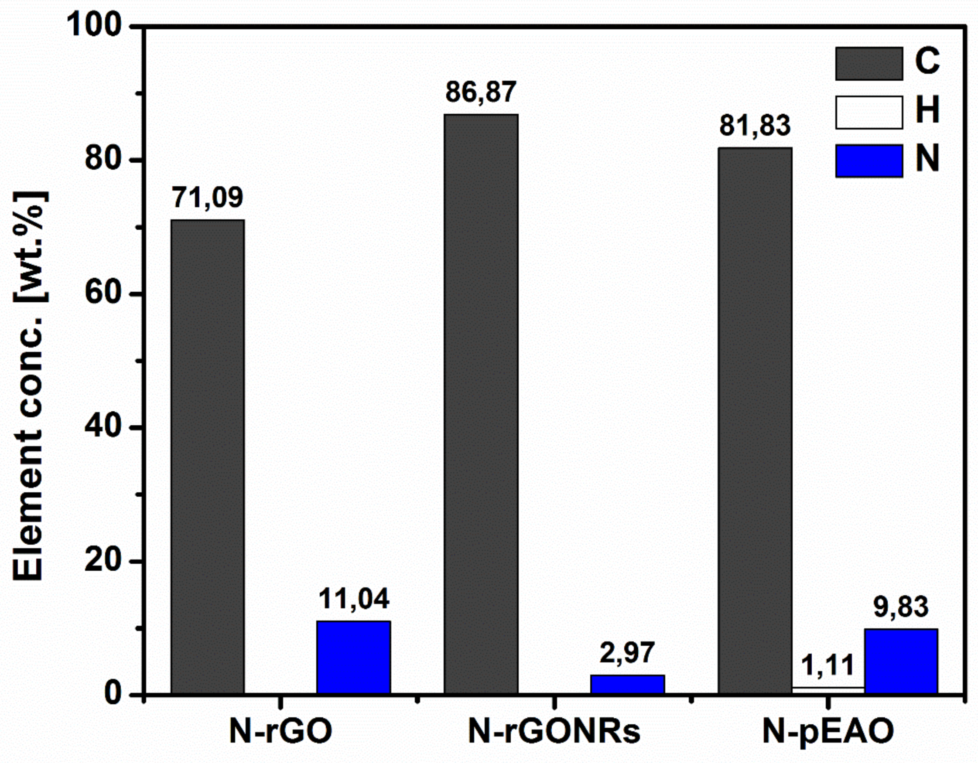

Figure S3. CHN elemental analysis of **N-rGO**, **N-rGONRs**, and **N-pEAO**.

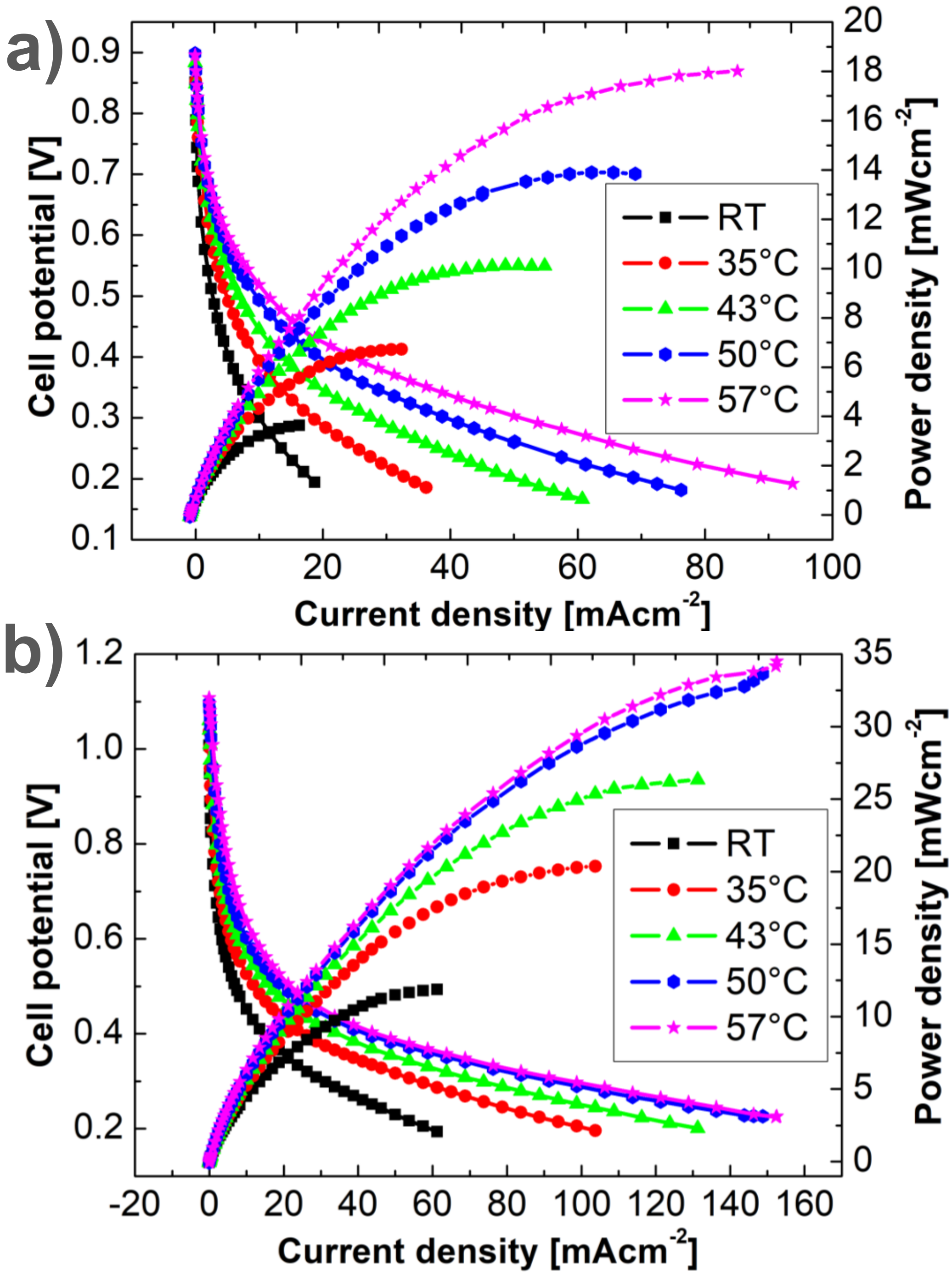

Figure S4. Typical DEAFC discharged cell voltage and power density of CS/**N-rGONRs** (0.07%) as a function of temperature in a) 1 M EtOH/1 M KOH and b) 3M EtOH / 5M KOH.

Table S1. $P_{max.}$ and related current density of CS membranes at 57 °C.

	**$P_{max.}$ 0.01 % [$mWcm^{-2}$]**	**$P_{max.}$ 0.04 % [$mWcm^{-2}$]**	**$P_{max.}$ 0.07 % [$mWcm^{-2}$]**
		**1 M EtOH/1 M KOH**	
CS/**N-rGO**	3.4 (9.69 $mAcm^{-2}$)	5.6 (23.01 $mAcm^{-2}$)	12.2 (62.81 $mAcm^{-2}$)
CS/**N-rGONRs**	10.7 (56.13 $mAcm^{-2}$)	10.9 (56.18 $mAcm^{-2}$)	18.0 (93.75 $mAcm^{-2}$)
CS/**N-pEAO**	7.4 (38.99 $mAcm^{-2}$)	10.5 (56.14 $mAcm^{-2}$)	12.1 (64.65 $mAcm^{-2}$)
		**3 M EtOH/5 M KOH**	
CS/**N-rGONRs**	28.9 (152.42 $mAcm^{-2}$)	22.9 (111.22 $mAcm^{-2}$)	34.5 (152.54 $mAcm^{-2}$)
CS/**N-pEAO**	20.5 (136.34 $mAcm^{-2}$)	24.2 (152. 22$mAcm^{-2}$)	21.8 (133.67 $mAcm^{-2}$)
